# Diagnosis of female 17α-hydroxylase deficiency after gonadectomy: a case report

**DOI:** 10.1186/s13256-019-2166-9

**Published:** 2019-07-30

**Authors:** Yukiko Mikami, Yasushi Takai, Mana Obata-Yasuoka, Ryo Kumagai, Hiroaki Yagyu, Kosuke Shigematsu, Haipeng Huang, Nozomi Uemura, Mamiko Shinsaka, Masahiro Saitoh, Kazunori Baba, Hiroyuki Seki

**Affiliations:** 1 Department of Obstetrics and Gynecology, Saitama Medical Center, Saitama Medical University, 1981 Kamoda, Kawagoe City, Saitama 350-3550 Japan; 2 Department of Obstetrics and Gynecology Faculty of Medicine, University of Tsukuba, Ibaraki, Japan; 3 Department of Endocrinology and Metabolism Tsukuba University Hospital Mito Clinical Education and Training Center, Mito Kyodo General Hospital, Ibaraki, Japan

**Keywords:** 17α-Hydroxylase, Genetic counseling, Hypertension, Sex development disorder, Rare disease

## Abstract

**Background:**

17α-Hydroxylase deficiency is a recessively inherited autosomal disease caused by mutations in the *CYP17A1* gene. It is a rare disease and accounts for approximately 1% of congenital adrenal cortex hyperplasias. Inhibition of 17α-hydroxylase causes low levels of cortisol and high levels of adrenocorticotropic hormone in the blood as well as excessive levels of mineralocorticoids that lead to hypertension and hypokalemia. Usually, the female patients are diagnosed with abnormality of the genitalia or extra genitalia, primary amenorrhea, or hypertension in puberty. We report a case of a 29-year-old woman who had undergone gonadectomy in her childhood due to complete androgen insensitivity syndrome and was diagnosed with 17α-hydroxylase deficiency in adulthood.

**Case presentation:**

Our patient was a Japanese female diagnosed with androgen insensitivity syndrome, and both gonadectomy and episioplasty were performed at the age of 11 years at the University of Tsukuba Hospital. Thereafter, she was transferred to our hospital at the age of 21 years for vaginoplasty. At the age of 25 years, she presented with hypertension followed by complicated hypokalemia at the age of 28 years. The captopril loading test and adrenocorticotropic hormone loading test of her adrenal steroidogenesis revealed primary aldosteronism. After sufficient genetic counseling, a genetic test was performed that identified her as having *CYP17A1* gene mutation.

**Conclusions:**

The differential diagnosis of disorders of sex development can be difficult at a young age without complete expression of the phenotype. However, diagnosis at a later age would change the treatment and prognosis of the disease; therefore, a genetic examination should be considered.

## Background

**Fig. 1:**
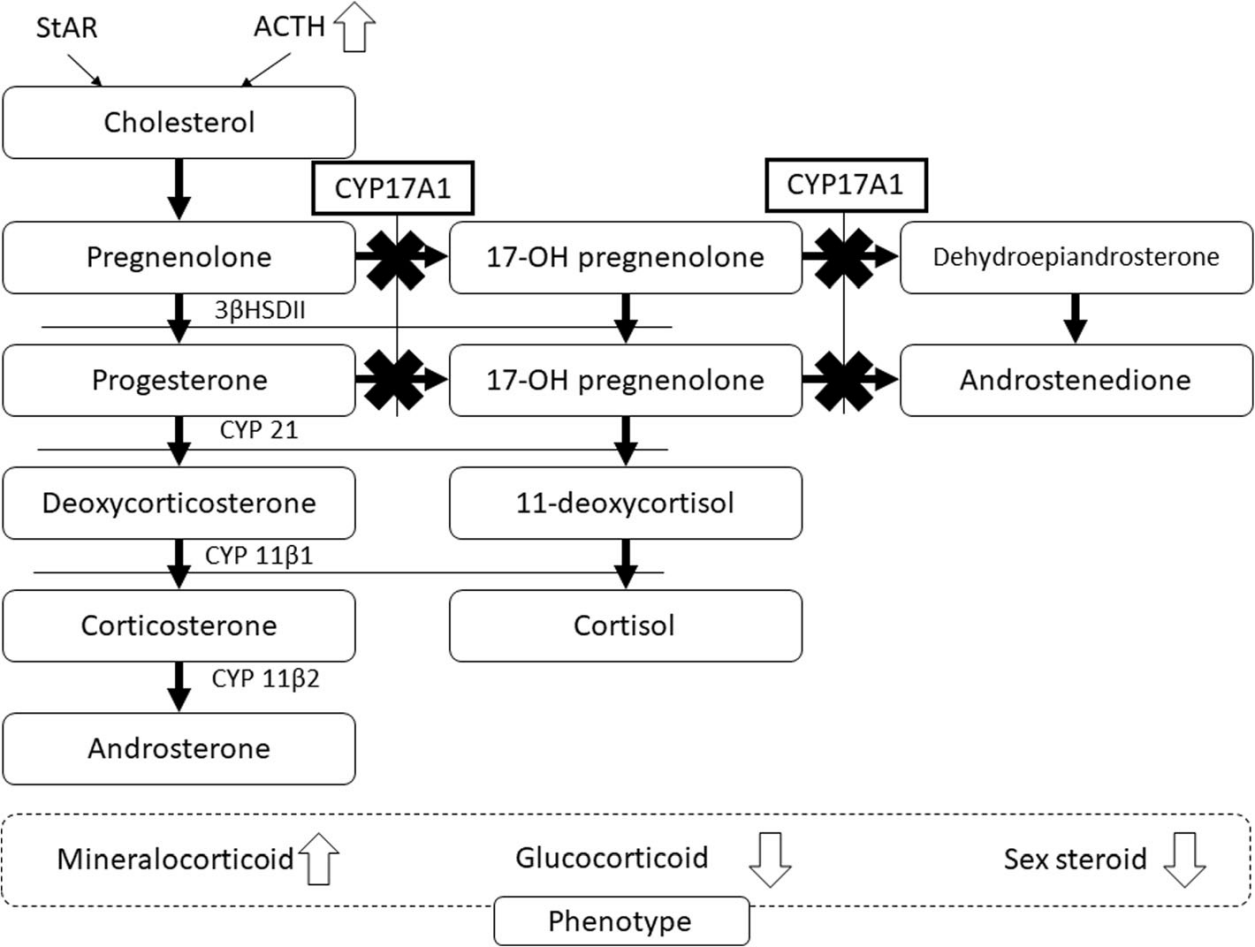
The metabolic pathway of adrenal cortex hormone. The variant of *CYP17A1* gene caused the deficiency of 17α-hydroxylase and metabolic disturbances from pregnenolone and progesterone to 17α-hydroxypregnenolone and 17α-hydroxyprogesterone. As a result, it causes excess of mineral corticoid and depression of glucocorticoid and sex steroid hormone

17α-Hydroxylase is an enzyme that converts pregnenolone and progesterone to 17α-hydroxypregnenolone and 17α-hydroxyprogesterone, which are the precursors of sex steroids and cortisol (Fig. 1). 17α-Hydroxylase deficiency (17OHD) is a recessively inherited autosomal disease caused by mutations in the *CYP17A1* gene [1]. It is a rare disease and accounts for approximately 1% of congenital adrenal cortex hyperplasias [2]. Patients with 17OHD typically have low blood levels of cortisol, estrogen, and androgens and a compensatory high blood level of adrenocorticotropic hormone (ACTH) [3]. Excessive levels of ACTH stimulate the production of 11-deoxycorticosterone and corticosterone, which have considerable mineralocorticoid activity, leading to extracellular volume expansion, hypertension, and hypokalemia [2, 3]. Deficiency of sex steroids causes primary amenorrhea in women [3] and feminization of external genitalia in men [4]. We report a case of a 29-year-old woman who had undergone gonadectomy in her childhood due to complete androgen insensitivity syndrome (AIS) and was diagnosed with 17OHD in adulthood.

## Case presentation

An 11-year-old Japanese girl was transferred to the University of Tsukuba Hospital for inspective examination for ambiguous genitalia. From visual inspection of the external genital area, a raised lesion with pigmentation and plicae that looked like fused labia were noted. Behind the lesion, there was an external urethral opening without an inlet for a vagina. At the time of consultation, the patient’s serum estradiol level was less than 8 pg/ml, serum testosterone level was less than 0.5 ng/dl, serum follicle-stimulating hormone (FSH) level was 5.6 mIU/ml, and serum luteinizing hormone (LH) level was 2.8 mIU/ml. Magnetic resonance imaging showed no uterine corpus or vagina in the pelvis and revealed small tumors in the bilateral inguinal position that looked like the testes. The patient’s karyotype was determined as 46,XY, without any electrolyte abnormality. She had no siblings, and her parents were both Japanese. There was also no family history of hypertension or electrolyte abnormalities.

Gonadectomy and episioplasty were performed on the basis of diagnosis of complete AIS. At the time of admission, the patient’s blood pressure was 111–116 mmHg systolic and 60–70 mmHg diastolic. The histopathological findings of the resected gonads revealed maturation arrest of the spermatogonia with no malignant lesion. The patient had continually taken conjugated estrogens since her gonadectomy.

After 10 years, the patient was transferred to our hospital for vaginoplasty. She had no electrolyte abnormality, and her blood pressure was 150–160 mmHg systolic and 100–110 mmHg diastolic at the time of admission. Therefore, the vaginoplasty was performed when she was 21 years old.

After 4 years of the vaginoplasty, the patient had started to take a hypotensive drug (nifedipine 10 mg/day); at the age of 28 years, she presented with hypokalemia complicated with hypertension. Therefore, she was hospitalized at Mito Kyodo General Hospital for a detailed examination. The patient’s hormone profile and the results of each loading test are shown in Tables 1 and 2. The results were indicative of primary aldosteronism. Abdominal computed tomography demonstrated a bilateral adrenal gland swelling. Because 17OHD was suspected, further evaluation and appropriate genetic counseling were recommended.

**Table 1:** Basal hormone profile of the patient

	Results	Reference range
ACTH (pg/dl)	138	7.2–63.3 (morning)
Cortisol (μg/dl)	2.39	6.24–18.0 (morning)
DHEA-S (μg/dl)	7	85–690 (male)
PAC (pg/ml)	199	29.9–159.0 (decubitus)
PRA (ng/ml/hr)	0.3	0.3–2.9 (decubitus)
ARR	663	< 200

*Abbreviations: ACTH* Adrenocorticotrophic hormone, *ARR* Plasma aldosterone concentration/plasma renin activity, *DHEA-S* Dehydroepiandrosterone sulfate, *PAC* Plasma aldosterone concentration, *PRA* Plasma renin activity

**Table 2:** Results of loading tests

Captopril suppressing test			
	Before	After 60 minutes	After 90 minutes
PAC (pg/ml)	225	157	130
PRA (ng/ml/hr)	0.2	0.4	0.3
ARR	1125	392.5	433.3
Furosemide stress test			
	Before	After 60 minutes	After 120 minutes
PAC (pg/ml)	198	182	175
PRA (ng/ml/hr)	0.3	0.4	0.4
Normal saline test			
	Before	After 240 minutes	
PAC (pg/ml)	174	99.4	
PRA (ng/ml/hr)	0.4	0.2	
Rapid ACTH stimulating test			
	Before	After 30 minutes	After 60 minutes
PAC (pg/ml)	172	220	217
PRA (ng/ml/hr)	0.2		
Cortisol	2.41	2.73	2.61

*Abbreviations: ACTH* Adrenocorticotrophic hormone, *ARR* Plasma aldosterone concentration/plasma renin activity, *DHEA-S* Dehydroepiandrosterone sulfate, *PAC* Plasma aldosterone concentration, *PRA* Plasma renin activity

Three months later, the patient and her family came to our hospital; after genetic counseling, the patient underwent the genetic test for the *CYP17A1* gene using multiplex PCR and targeted next-generation sequencing as described previously [5]. The genetic analysis showed a compound heterozygous deletion of phenylalanine (Phe) codon in exon 1 [c.160_162delTTC (p.Phe54del)] and heterozygous mutation in exon 6 [c.1118A>T (p.His373Leu)]. These genetic variations were reported by Miura *et al.* in Japan [6] and Kim *et al.* in Korea [3]. The composite *CYP17A1* mutant gene could be the cause of her disease. The genetic analysis of the patient’s mother revealed heterozygous mutation of exon 6 (c.1118A>T) and was acknowledged as the gene carrier. We could not perform genetic analysis of the patient’s father, because he could not be contacted at the time of testing, as her parents had divorced when she was a child. The blood pressure of the patient was controlled by a selective aldosterone blocker (eplerenone 50 mg/day) because she only showed symptoms of hyperaldosteronism and never showed any symptoms from the lower level of glucocorticoids, such as hypotension and dehydration. Fortunately, our patient did not have gender dysphoria identified socially as a female, and both she and her family accepted the results of the genetic examination.

## Discussion and conclusions

Congenital adrenal hyperplasia (CAH) is an autosomal recessive disorder due to a defect in any of the enzymes mediating steroidogenesis. 17OHD is a rare type of CAH that causes cortisol and sex hormone deficiency and aldosterone excess. The most common cause of CAH is 21-hydroxylase deficiency (21OHD), and neonatal screening programs for CAH are designed to detect only 21OHD [7]. It is reported that patients with 17OHD are usually diagnosed by the anomaly of external genitalia at birth or in childhood [8], lack of sexual development, or hypertension in puberty [2, 6, 9, 10]. The clinician whose practice involves young patients with hypertension and primary amenorrhea should be aware of CAH as a differential diagnosis. However, when the phenotypic abnormality is mild, as in our patient’s case, such cases are more likely to be diagnosed in adulthood [11, 12].

Our patient did not have hypertension or hypokalemia at the age of 11 years. Although the levels of FSH and LH suggested the onset of puberty, the level of sex steroids was low, which can be considered as an indicator of differential diagnosis. Additionally, various load testing techniques (captopril suppressing test, saline loading test, and furosemide stress test) should be considered to confirm primary aldosteronism even when the patients are normotensive.

Disorders of sex development (DSD) with 46,XY have an abdominal gonad that presents a high risk of tumor development (about 35%) [13]. However, the risk of tubular *in situ* neoplasia is reported to increase later in life, and ultrasonography can help in the identification of the gonads once their precise position is known [13]. Therefore, recent guidelines recommend gonadectomy after puberty [14]. In this case, the genetic examination should have been considered at the age of 11 years because the result would have been useful in determining an indication for gonadectomy and other necessary treatments. Furthermore, we must consider the gender identity of the patient. Fortunately, the current patient did not have gender dysphoria and identified socially as female, and both the patient and her family accepted the results of the genetic examination.

The purposes of the diagnosis of DSD, including genetic examinations, are to avoid errors in the identification of acute adrenal insufficiency, which is potentially life-threatening, and to predict the issues that may present, such as hypertension, obesity, and insulin resistance. For precise individual care, a multidisciplinary team that includes not only a gynecologist but also a pediatric endocrine specialist, a clinical geneticist, and a genetic counselor should perform physical phenotyping and hormonal examination along with the genetic testing [15].

In conclusion, the differential diagnosis of DSD may be difficult when the patient is younger without complete phenotypes. However, because a late diagnosis could result in different treatment and prognosis, a genetic examination should be considered. In addition, a multidisciplinary team should be involved in the patient’s treatment and in communication with the patient and the family.

## Data Availability

Not applicable.

## Abbreviations

17OHD: 17α-Hydroxylase deficiency
21OHD: 21-Hydroxylase deficiency
ACTH: Adrenocorticotropic hormone
AIS: Androgen insensitivity syndrome
ARR: Plasma aldosterone concentration/plasma renin activity
CAH: Congenital adrenal hyperplasia
DHEA-S: Dehydroepiandrosterone sulfate
DSD: Disorders of sex development
FSH: Follicle-stimulating hormone
LH: Luteinizing hormone
PAC: Plasma aldosterone concentration
PRA: Plasma renin activity

## References

[1] Xue LQ, Han B, Chen LB, Pan CM, Zhu H, Liu BL, *et al.* Identification of a novel mutation in *CYP17A1* gene. Transl Res. 2013;161(1):44–9. 10.1016/j.trsl.2012.08.007 23036723

[2] Xu S, Hu S, Yu X, Zhang M, Yang Y. 17αhydroxylase/17,20lyase deficiency in congenital adrenal hyperplasia: a case report. Mol Med Rep. 2017;15:339–44. 10.3892/mmr.2016.6029 PMC5355729 27959413

[3] Kim SM, Rhee JH. A case of 17α-hydroxylase deficiency. Clin Exp Reprod Med. 2015;42:72–6. 10.5653/cerm.2015.42.2.72 PMC4496435 26161337

[4] Auchus RJ. Steroid 17-hydroxylase and 17,20-lyase deficiencies, genetic and pharmacologic. J Steroid Biochem Mol Biol. 2017;165(Pt A):71–8. 10.1016/j.jsbmb.2016.02.002 PMC4976049 26862015

[5] Guzzetti C, Bizzarri C, Pisaneschi E, Mucciolo M, Bellacchio E, Ibba A, *et al.* Next-generation sequencing identifies different genetic defects in 2 patients with primary adrenal insufficiency and gonadotropin-independent precocious puberty. Horm Res Paediatr. 2018;90:203–11. 10.1159/000492496 30179867

[6] Miura K, Yasuda K, Yanase T, Yamakita N, Sasano H, Nawata H, *et al.* Mutation of cytochrome P-45017 alpha gene (CYP17) in a Japanese patient previously reported as having glucocorticoid-responsive hyperaldosteronism: with a review of Japanese patients with mutations of CYP17. J Clin Endocrinol Metab. 1996;81:3797–801. 10.1210/jcem.81.10.8855840 8855840

[7] Hinz L, Pacaud D, Kline G. Congenital adrenal hyperplasia causing hypertension: an illustrative review. J Hum Hypertens. 2018;32:150–7. 10.1038/s41371-017-0002-5 29255217

[8] Kastumata N, Fujiwara I, Ogawa H, Fujiwara K. Adrenal glands: genetic analysis of 17α hydroxylase deficiency that does not present the high blood pressure [in Japanese]. Clin Endocrinol. 2011;59:271–4.

[9] Lin D, Harikrishna JA, Moore CC, Jones KL, Miller WL. Missense mutation serine106—proline causes 17 alpha-hydroxylase deficiency. J Biol Chem. 1991;266:15992–8. 1714904

[10] Monno S, Takasu N. A new variant of 17 alpha-hydroxylase deficiency with hyperaldosteronism in two Japanese sisters [in Japanese]. Endocrinol Jpn. 1989;36:315–23. 10.1507/endocrj1954.36.315 2550210

[11] Mula-Abed WA, Pambinezhuth FB, Al-Kindi MK, Al-Busaidi NB, Al-Muslahi HN, Al-Lamki MA. Congenital adrenal hyperplasia due to 17-alpha-hydoxylase/17,20-lyase deficiency presenting with hypertension and pseudohermaphroditism: First Case Report from Oman. Oman Med J. 2014;29:55–9. 10.5001/omj.2014.12 PMC3910417 24498484

[12] Ueda Y, Watanabe T, Usui K, Kakita M, Nakatani R, Nakao K, *et al.* The genetic endocrinological analysis of untreated adult 17α hydroxylase deficiency patient [in Japanese]. Folia Endocrinol Jpn. 2014;90(Suppl):72–4.

[13] Wunsch L, Holterhus PM, Wessel L, Hiort O. Patients with disorders of sex development (DSD) at risk of gonadal tumour development: management based on laparoscopic biopsy and molecular diagnosis. BJU Int. 2012;110:E958–65. 10.1111/j.1464-410X.2012.11181.x 22540217

[14] Mouriquand PD, Gorduza DB, Gay CL, Meyer-Bahlburg HF, Baker L, Baskin LS, *et al.* Surgery in disorders of sex development (DSD) with a gender issue: if (why), when, and how? J Pediatr Urol. 2016;12:139–49. 10.1016/j.jpurol.2016.04.001 27132944

[15] Audi L, Ahmed SF, Krone N, Cools M, McElreavey K, Holterhus PM, *et al.* Genetics in endocrinology: approaches to molecular genetic diagnosis in the management of differences/disorders of sex development (DSD): position paper of EU COST Action BM 1303 'DSDnet'. Eur J Endocrinol. 2018;179:R197–06. 10.1530/EJE-18-0256 PMC6182188 30299888

